# Functional Connectivity Hypointensity of Middle Cingulate Gyrus and Thalamus in Age-Related Macular Degeneration Patients: A Resting-State Functional Magnetic Resonance Imaging Study

**DOI:** 10.3389/fnagi.2022.854758

**Published:** 2022-03-21

**Authors:** Ang Xiao, Hai-Jun Li, Qiu-Yu Li, Rong-Bin Liang, Hui-Ye Shu, Qian-Min Ge, Xu-Lin Liao, Yi-Cong Pan, Jie-Li Wu, Ting Su, Li-Juan Zhang, Qiong Zhou, Yi Shao

**Affiliations:** ^1^Department of Ophthalmology, The First Affiliated Hospital of Nanchang University, Nanchang, China; ^2^Department of PET Center and Medical Image Center, The First Affiliated Hospital of Nanchang University, Nanchang, China; ^3^Department of Ophthalmology and Visual Sciences, The Chinese University of Hong Kong, Shatin, Hong Kong SAR, China; ^4^Department of Ophthalmology, Xiang’an Hospital of Xiamen University, Fujian Provincial Key Laboratory of Ophthalmology and Visual Science, Eye Institute of Xiamen University, Xiamen University School of Medicine, Xiamen, China; ^5^Department of Ophthalmology, Massachusetts Eye and Ear, Harvard Medical School, Boston, MA, United States

**Keywords:** age-related macular degeneration, rs-fMRI, functional connectivity, middle cingulate gyrus, thalamus

## Abstract

**Objective:**

Age-related macular degeneration (AMD) causes visual damage and blindness globally. The purpose of this study was to investigate changes in functional connectivity (FC) in AMD patients using resting-state functional magnetic resonance imaging (rs-fMRI).

**Subjects and Methods:**

A total of 23 patients (12 male, 11 female) with AMD were enrolled to the AMD patients group (AMDs), and 17 healthy age-, sex-, and education-matched controls (9 male, 8 female) to the healthy controls group (HCs). All participants underwent rs-fMRI and mean FC values were compared between the two groups.

**Results:**

Significantly higher FC values were found in the inferior frontal gyrus (IFG), superior frontal gyrus (SFG), inferior parietal lobule (IPL), rectal gyrus (RTG), and superior parietal lobule (SPL) in AMDs compared with HCs. Conversely, FC values in the cerebellum posterior lobe (CPL), middle cingulate gyrus (MCG), medulla (MDL), cerebellum anterior lobe (CAL), and thalamus (TLM) were significantly lower in AMDs than in HCs.

**Conclusion:**

This study demonstrated FC abnormalities in many specific cerebral regions in AMD patients, and may provide new insights for exploration of potential pathophysiological mechanism of AMD-induced functional cerebral changes.

## Introduction

As a prevalent, chronic and progressive disease of the macula, age-related macular degeneration (AMD) is the leading cause of central vision impairment worldwide. The prevalence of AMD ranges from 6.8% in Asians to 12.3% in Europeans, is lower in Africans than in Europeans, but similar between Asians and Africans (Kawasaki et al., 2010; Laude et al., 2010; Wong et al., 2014). Major visual impairment occurs mainly in the late stages of AMD in one of two forms: neovascular (wet) AMD and geographic (dry) atrophy. Age is an important risk factor for AMD, other strong and consistent risk factors being darker iris pigmentation (Chakravarthy et al., 2010), previous cataract surgery (Cugati et al., 2006), cigarette smoking (Seddon et al., 1996), and obesity (Seddon et al., 2003). In clinical practice, fundus fluorescein angiography (FFA), optical coherence tomography, and fundus autofluorescence imaging are now extensively applied in diagnosis and to guide management of AMD (Lim et al., 2012). However, these examinations may not be suitable for patients with severe ocular media opacity or significant disease such as heart or renal failure. Advances have been made in disease detection and diagnosis allowing for rapid intervention, monitoring, and amelioration of the disease, improving prognosis, and evaluation.

Resting-state functional magnetic resonance imaging (rs-fMRI) is widely performed to assess cerebrum functional connectivity (FC), which is temporally correlated within resting state functional networks. rs-fMRI is increasingly applied to map the representation of cerebral function in many diseases, such as amyotrophic lateral sclerosis (Douaud et al., 2011), traumatic brain injury (Mayer et al., 2015), stroke (Puig et al., 2018), and Alzheimer’s disease (Zhao et al., 2020), and has proven valuable for characterizing and analyzing cerebral activity in the resting state (Damoiseaux et al., 2006; De Luca et al., 2006) and in task performance (Spreng and Grady, 2010). Based on the correlation between the anatomical structure and physiological functions of the retina and cerebrum (Wong et al., 2001b; Patton et al., 2005), the potential of retinopathy to provide indirect indicators of intracerebral lesions has attracted extensive attention (Fuller et al., 2001; Wong et al., 2001a,^2002^; Yatsuya et al., 2010). Abnormal spontaneous FC has been observed in ophthalmic diseases such as glaucoma (Li et al., 2017), amblyopia (Ding et al., 2013; Liang et al., 2017), and strabismus (Yan et al., 2019). The frontal, thalamic and temporal cerebral regions comprise the default mode network, which participates in memory, emotional, and cognitive functions (Raichle, 2010; Zhang and Raichle, 2010). Therefore, we hypothesize that FC is abnormal in AMD patients, and that relevant cognition-related or connectivity changes in visual areas may result in anxiety and depression.

**FIGURE 1:**
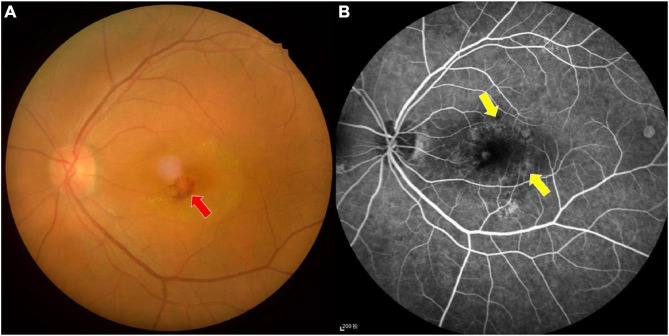
Fundus photograph **(A)** and fluorescence fundus angiography image **(B)** of age-related macular degeneration. The fundus photograph shows bleeding and exudation in the macular area, while the leakage of strong fluorescein spot and shadowing fluorescein at the macular area are shown in the fundus fluorescein angiography image. Red arrow indicates bleeding, and yellow arrows indicate the leakage.

To explore this possibility, rs-fMRI was used to measure cerebral FC, promoting an in-depth understanding of the potential neural mechanism of cerebrum visual pathway injury in patients with AMD (Figure 1), and allowing better evaluation and improved prognosis for patients.

## Materials and Methods

### Subjects

A total of 23 subjects with AMD (12 males, 11 females) were recruited to the AMD group (AMDs) at the First Affiliated Hospital of Nanchang University (Nanchang, China) according to the inclusion criteria described in previous publications (Seiple et al., 2005; Zuo et al., 2020). Information about the AMD patients is provided in section “Results.” Seventeen healthy controls (HCs) (9 male and 8 female subjects) without AMD were recruited to the HCs group. The two groups were matched for age, gender, handedness, educational level, and total intracranial volume. The inclusion criteria for HCs were as follows: (1) no history of AMD or other ocular disease; (2) no MRI contraindications; (3) no history of drug or alcohol abuse; and (4) no neurological or psychiatric diseases.

This study was conducted in accordance with the Declaration of Helsinki, and was approved by the Medical Ethics Committee of the First Affiliated Hospital of Nanchang University. All participants signed declarations of informed consent.

### Functional Magnetic Resonance Imaging Parameters

The MRI scans were performed using a 3-T MR scanner (Trio; Siemens, Munich, Germany). T1-weighted (T1W) gradient echo images were acquired using the following parameters: TR/TE 1,900/2.26 ms; gap 0.5 mm; slice thickness 1.0 mm; acquisition matrix 256 × 256; field of view 250 × 250 mm; and flip angle 9°. Functional images were processed using the following parameters: TR/TE 2,000/30 ms; gap 1.2 mm; slice thickness 4.0 mm; acquisition matrix 64 × 64; field of view 220 × 220 mm; flip angle 90°; number of axial slices 29. All participants were awake with eyes closed for the duration of the scan.

### Functional Magnetic Resonance Imaging Data Processing

The fMRI data were classified using MRIcro software,^1^ and processed using statistical parametric mapping software^2^ and the rs-fMRI Data Analysis Toolkit (REST^3^), using the Data Processing Assistant for Resting-State fMRI (DPARSF) software^4^ for resting-state fMRI. This methodology has been described previously (Chao-Gan and Yu-Feng, 2010; Huang et al., 2016). The first 10 volumes from each participant were eliminated to ensure the stability of signal values, and images were motion corrected. Data were smoothed using a Gaussian at full width half-maximum of 8 × 8 × 8 mm. Bandpass filtering (0.01–0.08 Hz) and image detrending were applied to reduce the influence of other factors that may lead to errors (Lowe et al., 1998).

### Functional Connectivity Analysis

The GIFT v3.0b toolbox^5^ was used for preprocessing analysis (Calhoun et al., 2001), and the rs-fMRI data preprocessing was performed using Data Processing & Analysis for Brain Imaging software (DPABI^6^) (Yan et al., 2016). The generalized linear model and one-way analysis of covariance were used to generate the FC maps. A previously published resting-state network template (Ding et al., 2011) was applied. This approach has been described in detail previously (Li et al., 2019).

### Statistical Analyses

SPSS software version 19.0 (IBM Corporation, Armonk, NY, United States) was used to analyze the processed data. Two-sample *t*-tests and Gaussian Random-Field theory were applied to correct for multiple comparisons. The correction parameters were set to voxel-level threshold of 0.005 and cluster-level threshold of 0.05, using a two-sided test. Age, gender, handedness and educational level were regression covariates. *P*-values < 0.05 were considered statistically significant. In addition, receiver operating characteristic (ROC) curves were generated to compare data from specific cerebral regions between the AMDs and HCs.

### Correlation Analysis

The Hospital Anxiety and Depression Scale (HADS) was completed by all participants. GraphPad Prism 8 software (GraphPad Inc., San Diego, CA, United States) was used for Pearson’s correlation analysis, and to evaluate and plot the linear correlation between HADS scores and mean FC signal values of the middle cingulate gyrus (MCG) and thalamus.

## Results

### Demographic and Clinical Characteristics

No significant differences in age (*P* = 0.819), weight (*P* = 0.876), or duration (*P* > 0.05) was found between AMDs and HCs. However, intraocular pressure (IOP) and binocular best corrected visual acuity were significantly different (*P* < 0.05) between the two groups. Details are shown in Table 1.

**TABLE 1:** Clinical characteristics of patients between AMDs and HCs.

Characteristics	AMDs	HCs	*t*-Value	*P*-Values
Male/female	12/11	9/8	0.124	0.972
Age (years)	55.72 ± 5.29	56.33 ± 5.62	−0.361	0.819
Weight (kg)	57.49 ± 7.72	58.41 ± 6.21	−0.484	0.876
Handedness (left/right)	0/23	0/17	NA	NA
Duration (years)	0.83 ± 0.49	NA	NA	NA
Best-corrected VA, left	0.15 ± 0.06*	1.06 ± 0.18	−2.732	0.031
Best-corrected VA, right	0.22 ± 0.09*	1.12 ± 0.34	−3.052	0.028
IOP, left	15.62 ± 3.27*	16.02 ± 4.11	−2.853	0.026
IOP, right	14.63 ± 3.25*	15.64 ± 3.46	−2.792	0.024

Independent t-tests comparing the two groups (*P < 0.05) represented statistically significant differences.

*AMDs, age-related macular degeneration group; HCs, healthy controls; NA, not applicable; VA, visual acuity; IOP, intraocular pressure.*

### Seed Regions of Interest

**FIGURE 2:**
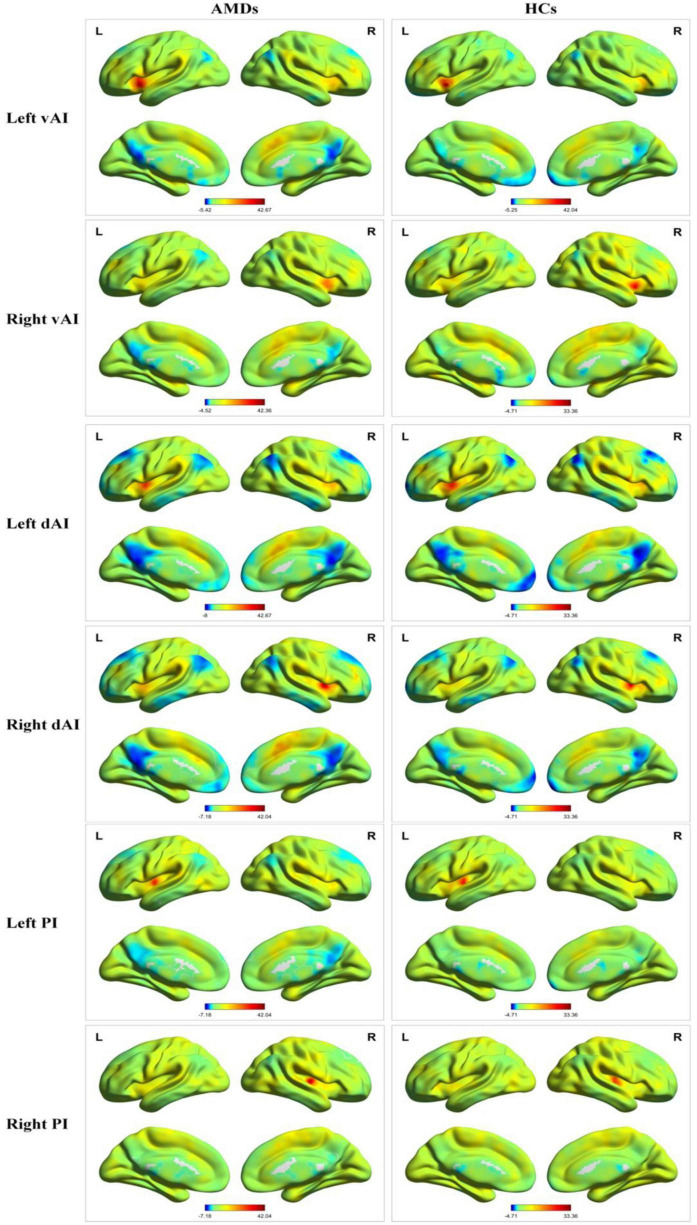
Resting-state functional connectivity patterns of insula subregions in AMDs and HCs. AMDs, age-related macular degeneration group; HCs, healthy controls; vAI, ventral anterior insula; dAI, dorsal anterior insula; PI, posterior insula; L, left; R, right.

The different insula subregions for resting-state FC patterns between the AMDs and HCs are shown in Figure 2. The ventral anterior insula (vAI) is chiefly connected with the limbic cortices and pregenual anterior cingulate mediating affective processes, while the dorsal anterior insula (dAI) connects with the dorsolateral prefrontal cortex and dorsal anterior cingulate cortex contributing to regulation of cognitive processes. Moreover, the posterior insula (PI) was predominantly connected with sensorimotor cortices. The three insular subregions were considered seed regions of interest to study the variability of resting-state FC in specific subregions of the insula for first episode schizophrenia and clinical high risk for psychosis (Li et al., 2019).

### Group Differences in Functional Connectivity

**FIGURE 3:**
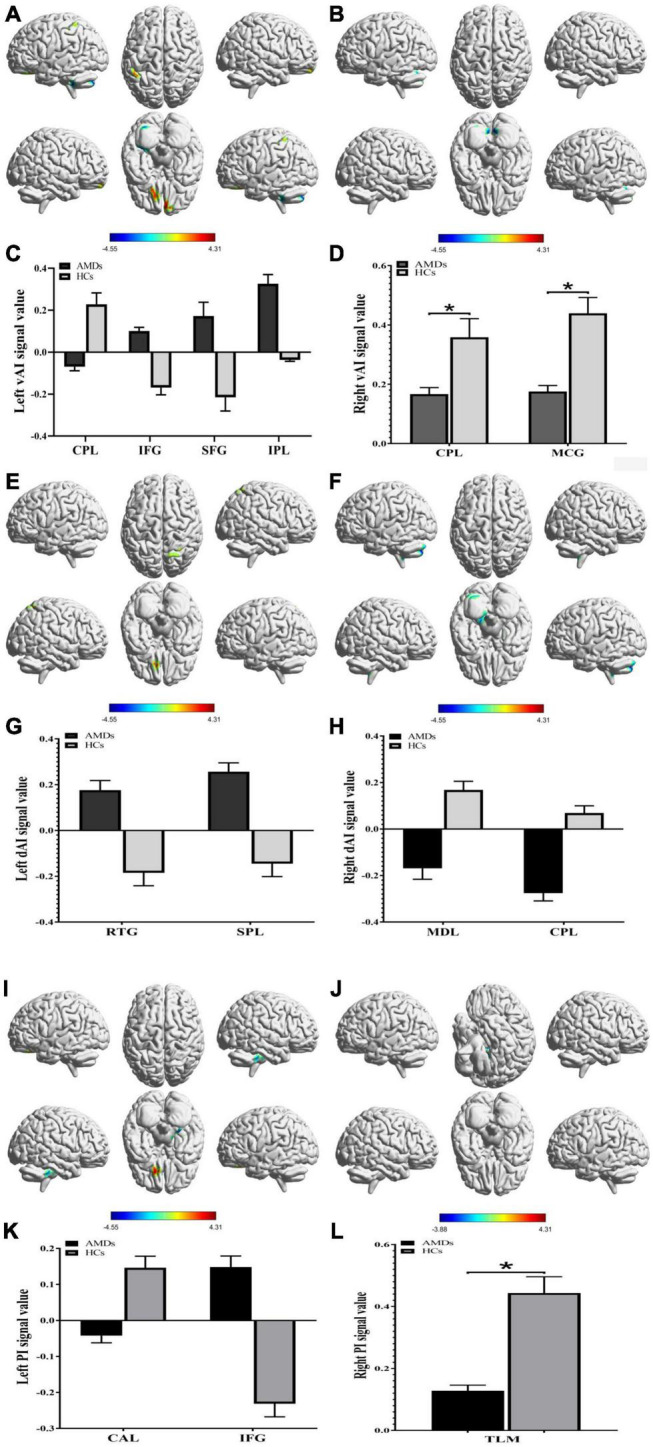
Functional connectivity group differences in insular subregions within different cerebral areas are shown **(A–L)**. **(A,C)**, **(B,D)**, **(E,G)**, **(F,H)**, **(I,K)**, and **(J,L)** show cerebral regions of altered FC in the left vAI, right vAI, left dAI, right dAI, left PI, and right PI, respectively. *P < 0.01. AMDs, age-related macular degeneration group; HCs, healthy controls; vAI, ventral anterior insula; dAI, dorsal anterior insula; PI, posterior insula; CPL, cerebellum posterior lobe; IFG, inferior frontal gyrus; SFG, superior frontal gyrus; IPL, inferior parietal lobule; MCG, middle cingulate gyrus; RTG, rectal gyrus; SPL, superior parietal lobule; MDL, medulla; CAL, cerebellum anterior lobe; TLM, thalamus.

The FC in specific cerebral regions are shown in Table 2 and Figure 3. We found that the mean FC values in inferior frontal gyrus (IFG), superior frontal gyrus (SFG), inferior parietal lobule (IPL), rectal gyrus (RTG), and superior parietal lobule (SPL) were statistically significantly higher in AMDs than in HCs, while values in cerebellum posterior lobe (CPL), MCG, medulla (MDL), cerebellum anterior lobe (CAL), and thalamus (TLM) were significantly lower in AMDs.

**TABLE 2:** Cerebral areas showing functional connectivity differences with insular subdivisions between AMDs and HCs.

Seed-ROIs	Cerebral areas	L/R	MNI coordinates			Number of voxels	*t*-Values
			*X*	*Y*	*Z*		
**Left vAI**							
	Cerebellum posterior lobe	L	−30	−75	−42	43	−4.547
	Inferior frontal gyrus	L	−12	42	−30	65	4.309
	Superior frontal gyrus	R	12	60	−12	73	4.122
	Inferior parietal lobule	L	−45	−36	42	72	3.945
**Right vAI**							
	Cerebellum posterior lobe	L/R	6	−75	33	65	−3.478
	Middle cingulate gyrus	L	−9	−12	36	50	−3.609
**Left dAI**							
	Rectal gyrus	L	−6	36	−27	40	3.986
	Superior parietal lobule	R	33	−63	66	46	5.196
**Right dAI**							
	Medulla	L	−6	−48	−51	42	−3.478
	Cerebellum posterior lobe	L	−24	−75	−39	68	−4.632
**Left PI**							
	Cerebellum anterior lobe	R	30	−30	−39	42	−3.908
	Inferior frontal gyrus	L	−12	36	−30	61	4.419
**Right PI**							
	Thalamus	R	12	−30	0	50	−3.875

*Voxel level P < 0.01, AlphaSim corrected.*

*AMDs, age-related macular degeneration group; HCs, healthy controls; L/R, left/right; vAI, ventral anterior insula; dAI, dorsal anterior insula; PI, posterior insula.*

### Receiver Operating Characteristic Curve

**FIGURE 4:**
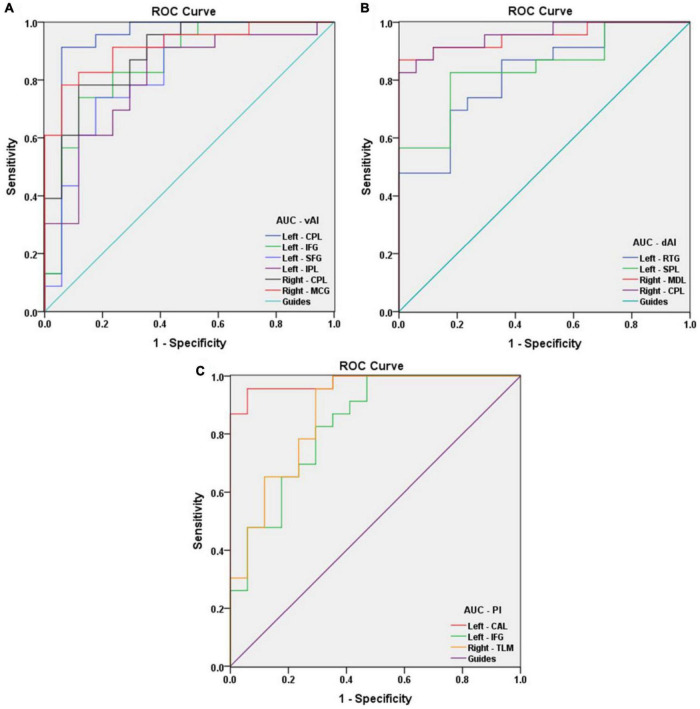
Receiver operating characteristic curve analysis of the mean FC values for the specific cerebral regions. **(A)** The area under the ROC curve of FC values were presented as follows: the CPL (0.934, 95% CI: 0.833–1.000), IFG (0.854, 95% CI: 0.730–0.978), SFG (0.831, 95% CI: 0.696–0.966), and IPL (0.806, 95% CI: 0.666–0.945) in left vAI, CPL (0.890, 95% CI: 0.790–0.990) and MCG (0.916, 95% CI: 0.830–1.000) in right vAI. **(B)** The AUCs of FC values in dAI were as follows: RTG (0.821, 95% CI: 0.693–0.949) and SPL (0.841, 95% CI: 0.719–0.964) in left dAI, MDL (0.951, 95% CI: 0.886–1.000) and CPL (0.957, 95% CI: 0.899–1.000) in right dAI. **(C)** The AUCs of FC values in PI were as follows: CAL (0.980, 95% CI: 0.944–1.000) and IFG (0.834, 95% CI: 0.705–0.962) in left PI, TLM (0.872, 95% CI: 0.758–0.987) in right PI. vAI, ventral anterior insula; dAI, dorsal anterior insula; PI, posterior insula; CPL, cerebellum posterior lobe; IFG, inferior frontal gyrus; SFG, superior frontal gyrus; IPL, inferior parietal lobule; MCG, middle cingulate gyrus; RTG, rectal gyrus; SPL, superior parietal lobule; MDL, medulla; CAL, cerebellum anterior lobe; TLM, thalamus.

Receiver operating characteristic curve analysis was used to verify differences and to explore whether FC values of specific cerebral regions have potential as biomarkers to differentiate patients with and without AMD. The individual areas under the curves (AUCs) of FC values within the range of regions are as follows: left vAI CPL (0.934, *P* < 0.001), IFG (0.854, *P* < 0.001), SFG (0.831, *P* < 0.001), and IPL (0.806, *P* = 0.001); right vAI CPL (0.890, *P* < 0.001) and MCG (0.916, *P* < 0.001) (Figure 4A); left dAI RTG (0.821, *P* = 0.001) and SPL (0.841, *P* < 0.001); right dAI MDL (0.951, *P* < 0.001) and CPL (0.957, *P* < 0.001) (Figure 4B); left PI CAL (0.980, *P* < 0.001) and IFG (0.834, *P* < 0.001); right PI TLM (0.872, *P* < 0.001) (Figure 4C). These findings indicate that the mean FC values of specific cerebral regions can accurately distinguish AMDs from HCs, and may be applied as diagnostic biomarkers.

### Correlation Analysis

**FIGURE 5:**
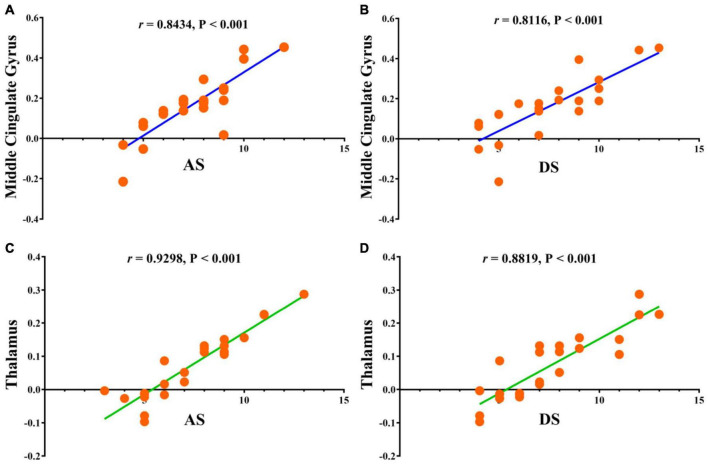
Correlations between the clinical behaviors and FC values in middle cingulate gyrus and thalamus. **(A)** The anxiety scores showed a positive correlation with FC values in middle cingulate gyrus (0.8434, 95% CI: 0.6610–0.9317); **(B)** the depression scores showed a positive correlation with FC values in middle cingulate gyrus (0.8116, 95% CI: 0.6002–0.9170); **(C)** the anxiety scores showed a positive correlation with FC values in thalamus (0.9298, 95% CI: 0.8392–0.9702); **(D)** the depression scores showed a positive correlation with FC values in thalamus (0.8819, 95% CI: 0.7380–0.9491). FC, functional connectivity; AS, anxiety scores; DS, depression scores.

Statistically significant positive correlations were found between HADS scores and overall FC values in the MCG (*r* = 0.8434, *P* < 0.0001 for anxiety and *r* = 0.8116, *P* < 0.0001 for depression; Figures 5A,B), and thalamus (*r* = 0.9298, *P* < 0.0001 for anxiety and *r* = 0.8819, *P* < 0.0001 for depression; Figures 5C,D) in AMDs.

## Discussion

**FIGURE 6:**
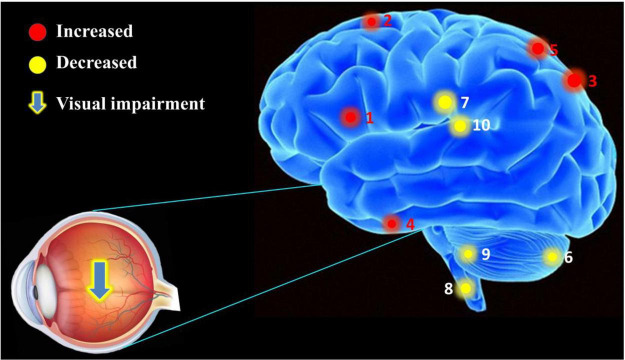
The mean FC values of cerebrum in AMD participants. Compared with HCs, the AMDs showed abnormal signals in specific cerebral regions as followed: 1. inferior frontal gyrus (left vAI, *t* = 4.309; left PI, *t* = 4.419), 2. superior frontal gyrus (*t* = 4.122), 3. inferior parietal lobule (*t* = 3.945), 4. rectal gyrus (*t* = 3.986), 5. superior parietal lobule (*t* = 5.196), 6. cerebellum posterior lobe (left vAI, *t* = –4.547; right vAI, *t* = –3.478; right dVI, *t* = –4.632), 7. middle cingulate gyrus (*t* = –3.609), 8. medulla (*t* = –3.478), 9. cerebellum anterior lobe (*t* = –3.908), and 10. thalamus (*t* = –3.875).

Foveal scotoma due to macular photoreceptor atrophy in AMD has caused vision loss and blindness for a large number of individuals globally, particularly in developed countries (Rosengarth et al., 2013; Wong et al., 2014). Previous studies in animals (Kaas et al., 1990; Darian-Smith and Gilbert, 1995; Giannikopoulos and Eysel, 2006) and humans (Pascual-Leone et al., 2005; Liu et al., 2007) have demonstrated altered cerebral functions in response to reduced visual input, but the extent of cerebral changes associated with AMD remains unclear and has attracted the attention of many researchers. Our study has indicated that FC values are significantly increased in the IFG, SFG, IPL, RTG, and SPL, and decreased in the CPL, MCG, MDL, CAL, and TLM in AMDs compared with HCs (Figure 6). These findings may reflect compensatory changes supporting cerebral performance in AMD patients with vision loss.

Previous MRI research on the impact of AMD on cerebral regions is shown in Table 3 and the underlying functions of specific areas of the cerebrum are shown in Table 4. We could hypothesize that AMD not only causes changes in cerebral FC patterns affecting visual pathways, language, cognitive and memory, but also strengthens internetwork connections *via* a loss of inhibitory signals that accompany visual stimulation or contribute to recruitment of new networks to support and complete visually mediated tasks. Whitson et al. (2015) found relatively high resting-state FC values in AMD patients in the IFG, superior temporal gyrus (STG), inferior parietal lobe (IPL), superior parietal lobe (SPL), supramarginal gyrus (SMG), supplementary motor area (SMA), and precentral gyrus (preCG). They also found high connectivity between SMA and SPL as well as SMA, IPL and IFG which are implicated in motor/visuospatial function, with strong connectivity within the reference links and default mode network compared to HCs. Furthermore, the AMDs showed stronger relationships between connectivity and memory performance in the inferior and medial temporal gyri, temporal pole, IFG, SFG, MFG, posterior cingulate cortex, and medial prefrontal cortex compared to control participants, while the resting default mode network in the bilateral posterior cingulate cortex, anterior cingulate cortices, and precuneus were similar in these groups (Zuo et al., 2020). One study indicates that AMDs exhibit increased cerebral activation in a widely distributed cortical network including SPL, IPL, the frontal eye fields, and the prefrontal cortex (Szlyk and Little, 2009). These results directly and indirectly support our findings that the mean FC values in IFG, SFG, IPL, RTG, and SPL are significantly higher in AMDs than in HCs, suggesting that a positively FC was correlated with these specific cerebral regions involved in the regulatory mechanism of AMD to achieve adaptability and plasticity in various functions such as vision, cognition, and memory.

**TABLE 3:** Current research status of fMRI and AMD in specific cerebral regions.

Author (Y)	Average age (Y)	Number (P/HC, M/F)	fMRI	Objective of cerebrum function	Cerebral regions
Little et al., 2008	AMDs, 55–83; Control, 22–78	18 (6/12, 8/10)	Yes	Cortical networks underling oculomotor function	**Increased:** preFC, intraparietal sulci, FEFs, supplementary eye fields; **Decreased:** visual cortex (MT/V5, V2/V3, and V1).
Rosengarth et al., 2013	AMDs, 55–84; Control, 51–83	16 (9/7, 6/10)	Yes	Training-related changes in cerebellum	**Increased:** fusiform gyrus, ITG, and lateral occipital cortex; **No differences:** visual area (V1, V2, and V3).
Zuo et al., 2020	AMDs, 75.3 ± 8.9; Control, 74.5 ± 7.2	83 (42/41, 40/43)	Yes	Quantify the strength of functional connectivity	**Increased:** ITG, MTG, temporal pole, IFG, SFG, MFG, PCC, and medial preFC; **No differences:** bilateral PCC, ACC, and precuneus.
Whitson et al., 2015	AMDs, 79.9 ± 7.5; Control, 68.3 ± 3.4	23 (7/16, 9/14)	Yes	Functional connectivity and phonemic fluency	**Increased:** left IFG, left STG, bilateral IPL, right SPL, right SMG, right SMA, and right precentral gyrus.
Szlyk and Little, 2009	AMDs, 55–83; Control, 54–78	12 (6/6, 5/7)	Yes	Cortical networks underling word recognition and processing	**Increased:** supplementary motor regions, FEFs, IPL and SPL, preFC; **Decreased:** SPL and IPL, primary and secondary visual cortices, FEFs, bilaterally SPL, supplementary motor regions and eye fields, and LFG.

*Y, year; P, patient; HC, healthy control; M, male; F, female; AMDs, age-related macular degeneration patients; ITG, inferior temporal gyrus; MTG, medial temporal gyrus; IFG, inferior frontal gyrus; SFG, superior frontal gyrus; MFG, middle frontal gyrus; PCC, posterior cingulate cortex; preFC, prefrontal cortex; ACC, anterior cingulate cortices; STG, superior temporal gyrus; IPL, inferior parietal lobe; SMG, supramarginal gyrus; FEFs, frontal eye fields; SMA, supplementary motor area; SPL, superior parietal lobe; LFG, left fusiform gyrus.*

**TABLE 4:** Alternation of cerebral regions and its potential effects.

Cerebral regions	Experimental results	Cerebral functions	Anticipate effects
Cerebellum posterior lobe	AMDs < HCs	Coordinate sensory and motor functions, and participate in higher cognitive functions	Dyskinesia and difficulty in fine motion
Inferior frontal gyrus	AMDs > HCs	Involved in language processing and cognitive functions	Bipolar disorder
Superior frontal gyrus	AMDs > HCs	Involved in cognitive and motor control, and the execution of working memory	Parkinson’s disease and motor aphasia
Inferior parietal lobule	AMDs > HCs	Involved in the processing of various sensory, perceptual and cognitive functions	Gerstmann’s syndrome and Schizophrenia
Middle cingulate gyrus	AMDs < HCs	Manage emotion, cognition, and movement, and integrate visual information	Affective and cognitive dysfunction, visual function abnormals
Rectal gyrus	AMDs > HCs	Decision making, reward processing, planning, and reasoning	Epilepsy
Superior parietal lobule	AMDs > HCs	Participates in somatosensory and working memory, and coordinates visual and motor functions	Cortical sensory disorders, such as loss of position, entity, and recognition
Medulla	AMDs < HCs	Control all non-conscious daily activities	Partial sensory loss, hemiplegia, and hemianopia
Cerebellum anterior lobe	AMDs < HCs	mediating unconscious proprioception, and regulate muscular tension	Cerebellar ataxia
Thalamus	AMDs < HCs	Sensory processing, memory function, emotion, associated with visual function	Emotional problems, endocrine disease, and visual dysfunction

In addition, one study showed significantly increased gray and white matter in the CPL during a period of oculomotor training in AMD compared with controls (Rosengarth et al., 2013), while no difference was found in white matter of the cingulum hippocampus, cingulate, or the thalamus (Yoshimine et al., 2018). In addition, volumetric reductions were found in the optic radiations, lateral geniculate bodies and visual cortex in AMD patients, as the white matter in frontal lobe was decreased in AMD but not in juvenile macular degeneration (Hernowo et al., 2014). The activation with significant clusters showed marked reduction in the parietal lobules in AMD patients (Little et al., 2008). Since the lateral geniculate body, optic radiation, and visual cortex are vital parts of the visual pathways, AMD patients may have changes in these specific areas. Szlyk and Little (2009) found decreased activation in the left SPL and IPL, primary and secondary visual cortices, frontal eye fields, bilateral superior parietal regions, supplementary motor regions, eye fields, and left fusiform gyrus in AMD patients relative to controls. However, the results of the present study differ from the above findings due to differences in race, geographical region, inter-individual variations and image data processing, but can fully supplement their research. Previous studies (Hernowo et al., 2014; Prins et al., 2016a,b) reported reduced cortical volume and abnormality of white matter in the visual cortex in AMD. The results of these studies suggest that AMD may cause widespread changes in cerebral regions and suggest a strong association between AMD and changes in specific cerebral regions, and their corresponding major effects, and are further supported by the current work.

**FIGURE 7:**
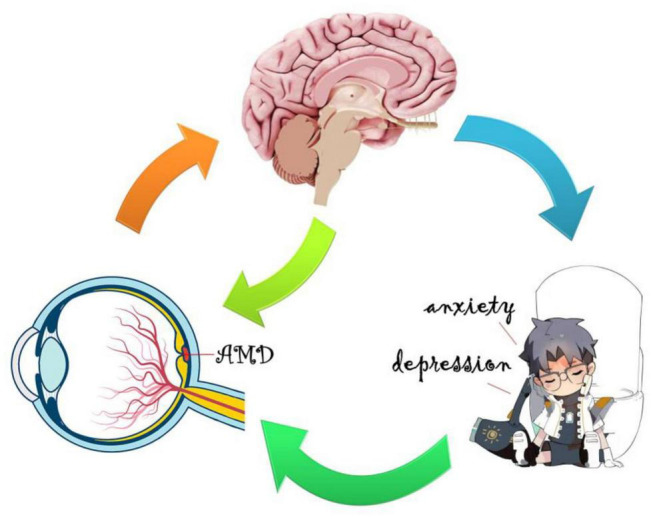
Relationship between FC values and emotional status. The mean FC values presented obvious abnormalities in many specific cerebral regions of AMD patients in contrast to healthy controls, and AMD patients appear to be more prone to anxiety and depression.

Whether changes in cerebral activity are consequential or adaptive in AMD and the exact mechanism underpinning them remains unclear. Apoptosis of retinal nerve cells, especially in the macular area, could affect changes in cerebral tissue properties through transsynaptic degeneration in AMD (Haak et al., 2016; Prins et al., 2016a). This would lead to decreased visual signaling from the defective macula and behavioral factors correlated with loss of visually dependent activities, such as social interaction and reading (Zuo et al., 2020). The frontal lobe, cingulate gyrus, temporal gyrus, and thalamus are involved in cognitive, emotional, and memory functions (Critchley et al., 2004; Seitz et al., 2006; Raichle, 2010; Zhang and Raichle, 2010). Lesions of these cerebral regions are connected with social and emotional behavior, and could lead to anxiety and depression. The AMD group in this study showed significant correlations between connectivity in cerebral regions and HADS scores, indicating that anxiety and depression scores are linked with overall FC values, and that abnormal neural electrical activity may occur in brain regions associated with emotional activity (Figure 7). Moreover, AMD may result in specific cerebral regional changes through a variety of mechanisms, including the loss of cognitive stimulation (a consequence of sensory disorder), adaptive restructuring of visual pathways, decreased feedback regulatory signals from visual cortical regions, or by increased metabolic demand in specific cerebral regions, which would present alterations in FC and diminished cognitive ability (Whitson et al., 2015). These changes may promote functional cerebral reorganization in the fronto-parietal control networks (Zhuang et al., 2018) and primary visual cortex (Masuda et al., 2008) in AMD to improve vision and prognosis. The mechanism of cerebral regional changes caused by AMD is a complex process involving many different brain regions and requires more in-depth and comprehensive research.

**FIGURE 8:**
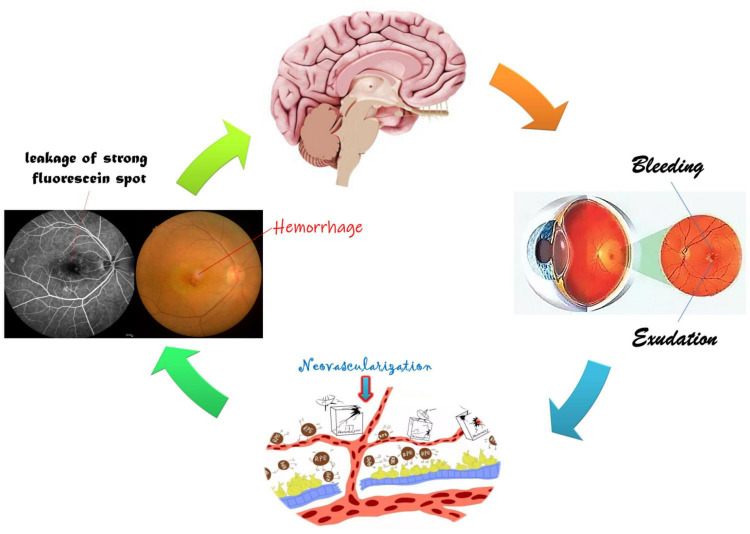
Relationship between FC and clinical manifestation of AMD. The retinal macular is stimulated by a variety of growth factors and inflammatory factors resulting in neovascularization, hemorrhage, and exudation, further leading to visual impairment and changes in specific cerebral regions.

Based on the above studies (Table 4 and Figure 8), it could be suggested that AMD patients have abnormalities in several cerebral regions. The current study has some limitations. A cross-sectional and observational approach was adopted in this study, and the sample size was small, making it difficult to observe the possible development of cognitive impairment and fMRI changes associated with AMD, so we have not drawn firm conclusions about causality of the described relationships. Therefore, an in-depth, comprehensive and systematic study is needed to elucidate the mechanisms and correlations of AMD-induced changes in brain regions.

## Conclusion

Our study demonstrated abnormal FC values in specific cerebral regions of AMD patients. These findings can not only supplement the theoretical basis for research on the mechanisms of AMD nerve injury and repair process, but also form a basis for further exploration of the potential pathophysiological mechanisms of AMD-induced functional changes in cerebral regions.

## Data Availability Statement

The original contributions presented in the study are included in the article/supplementary material, further inquiries can be directed to the corresponding author/s.

## Ethics Statement

The studies involving human participants were reviewed and all research methods were approved by the Committee of the Medical Ethics of the First Affiliated Hospital of Nanchang University and were in accordance with the 1964 Helsinki declaration and its later amendments or comparable ethical standards. The patients/participants provided their written informed consent to participate in this study.

## Author Contributions

All authors listed have made a substantial, direct, and intellectual contribution to the work, and approved it for publication.

## Conflict of Interest

The authors declare that the research was conducted in the absence of any commercial or financial relationships that could be construed as a potential conflict of interest.

## Publisher’s Note

All claims expressed in this article are solely those of the authors and do not necessarily represent those of their affiliated organizations, or those of the publisher, the editors and the reviewers. Any product that may be evaluated in this article, or claim that may be made by its manufacturer, is not guaranteed or endorsed by the publisher.

## Footnotes

1
www.MRIcro.com

2
http://www.fil.ion.ucl.ac.uk/spm/

3
http://www.restfmri.net

4
http://rfmri.org/DPARSF

5
http://icatb.sourceforge.net/

6
http://www.rfmri.org/dpabi
